# Infra-red photoresponse of mesoscopic NiO-based solar cells sensitized with PbS quantum dot

**DOI:** 10.1038/srep24908

**Published:** 2016-04-29

**Authors:** Mahfoudh Raissi, Yann Pellegrin, Stéphane Jobic, Mohammed Boujtita, Fabrice Odobel

**Affiliations:** 1 CEISAM, Chimie Et Interdisciplinarité, Synthèse, Analyse, Modélisation CNRS, UMR CNRS 6230, UFR des Sciences et des Techniques 2, rue de la Houssinière-BP 92208; 44322 NANTES Cedex 3 France; 2 Institut des Matériaux Jean Rouxel, Université de Nantes, CNRS, 2, rue de la Houssinière, BP 32229, 44322 Nantes cedex 03, France

## Abstract

Sensitized NiO based photocathode is a new field of investigation with increasing scientific interest in relation with the development of tandem dye-sensitized solar cells (photovoltaic) and dye-sensitized photoelectrosynthetic cells (solar fuel). We demonstrate herein that PbS quantum dots (QDs) represent promising inorganic sensitizers for NiO-based quantum dot-sensitized solar cells (QDSSCs). The solar cell sensitized with PbS quantum dot exhibits significantly higher photoconversion efficiency than solar cells sensitized with a classical and efficient molecular sensitizer (P1 dye = 4-(Bis-{4-[5-(2,2-dicyano-vinyl)-thiophene-2-yl]-phenyl}-amino)-benzoic acid). Furthermore, the system features an IPCE (Incident Photon-to-Current Efficiency) spectrum that spreads into the infra-red region, reaching operating wavelengths of 950 nm. The QDSSC photoelectrochemical device works with the complexes *tris*(4,4′-di*tert*-butyl-2,2′-bipyridine)cobalt(III/II) redox mediators, underscoring the formation of a long-lived charge-separated state. The electrochemical impedance spectrocopy measurements are consistent with a high packing of the QDs upon the NiO surface, the high density of which limits the access of the electrolyte and results in favorable light absorption cross-sections and a significant hole lifetime. These notable results highlight the potential of NiO-based photocathodes sensitized with quantum dots for accessing and exploiting the low-energy part of the solar spectrum in photovoltaic and photocatalysis applications.

Sensitization of mesoscopic NiO films with molecular dyes has attracted scientific interest during the past decade because these photocathodes represent the key component of tandem dye-sensitized solar cells (DSSCs) and tandem dye-sensitized photoelectrosynthetic cells^1^,^2^,^3^. In these fields, quantum dots (QDs) have been far less investigated than molecular sensitizers despite having demonstrated significant promise as light collectors in other photovoltaic technologies, including Schottky type solar cells^4^,^5^ and conventional TiO_2_-based n-QDSSCs^6^,^7^. QDs are particularly well-suited as light collectors for p-type sensitized solar cells (p-DSSC) due to their unique and valuable properties, which we shall briefly summarize. Firstly, their high absorption coefficients render them excellent candidates for reaching high optical density upon thin NiO layers, given the short hole diffusion length (ca. 3 μm) in such devices^3^. Secondly, inorganic absorbers such as metal chalcogenides display higher photostability compared to molecular dyes, and are easier and faster to synthesize than the latter^6^,^8^. Lastly, it bears mentioning that their tunable bandgaps allow for the design of efficient IR absorbers–PbS, PbSe, and CuInSe_2_ among others–with minimal effort in accordance with well-established colloidal synthetic methods^9^,^10^. The ability to exploit infrared photons is highly valuable, not only for the improvement of the intrinsic solar photoconversion efficiency (PCE) of the photocathodes, but also for their implemention into tandem devices in order to harvest the remaining incoming energy not absorbed by the photoanode. Similar to molecular sensitizers, the nanocrystal of QD can be grafted onto the semiconductor (SC) surface by means of a carboxylic acid anchoring group *via* a bifunctional linker, typically mercaptopropionic acid (MPA), following well-documented *ex-situ* linker-assisted methodologies^6^,^7^. Other deposition techniques such as the successive ion layer absorption and reaction (SILAR), Pulsed Laser Deposition (PLD)^11^,^12^,^13^ and chemical bath deposition (CBD) are routinely used *in situ* methods to prepare QDSSCs^7^. So far, CdS^14^,^15^,^16^ and CdSe^17^,^18^,^19^,^20^,^21^ are the most investigated metal chalcogenides in NiO-based p-QDSSCs, although Cu_2_S^22^, CdTeO_3_^23^ and more recently CuInS_2_ were reported^24^,^25^. Frank and co-workers^26^, have reported very fast hole transport times for NiO film sensitized with CdS QDs, resulting in an almost quantitative charge collection efficiency. Pullerits and co-workers^21^,^27^ have shown that hole trapping by the defects on CdSe QDs diminishes the hole injection efficiency (ϕ_inj_) in NiO valence band, but that the presence of a ZnS shell can passivate these traps and enhance ϕ_inj_. Wang and co-workers^16^ have reported the best-performing p-QDSSC by successive deposition of CdSe over CdS QDs on a NiO nanostructured film. The relative position of the valence and conduction band potentials of the overlaid CdS/CdSe QDs decreases the charge recombination reactions of the injected hole with the electron in the QD or with the electrolyte, reducing the major losses associated with undesirable pathways.

Recently, NiO films sensitized with CdSe quantum dots have also been successfully implemented in photoelectrocatalytic solar cells for hydrogen production^17^,^28^,^29^,^30^. These aforementioned attractions and properties of QDs have thus prompted our efforts to assess the possibility of using a low bandgap QD, such as PbS, to prepare efficient NiO-based photoelectrochemical devices. In this study, we describe the fabrication of NiO-based QDSSC using PbS QDs (Fig. 1) with a cobalt electrolyte (*tris*(4,4′- di*tert*-butyl-2,2′-bipyridine)cobalt(III/II) redox couple structure shown in Fig. S6). We find that the system delivers up to 5.27 mA/cm^2^ of short-circuit current density with an IPCE extending well into the IR range exceeding 950 nm–results that highlight the compelling potential of p-QDSSCs.

## Results

### Preparation and characterization of PbS quantum dots

Highly monodispersed oleic acid (OA) capped PbS quantum dots with average diameters of 2.9 nm were prepared according to the literature with the hot injection method^9^. It is well-accepted that QDs contain many intragap states, which act as traps and quench luminescence^4^,^5^,^8^. Halide anions, and iodide in particular, can efficiently passivate the surface traps on PbS QDs, resulting in a longer-lived exciton (electron/hole pair) lifetime^4^,^5^. It has also been clearly demonstrated that the passivation of the traps in QDs is a crucial factor for highly performing solar cells^4^,^5^. In addition, the insulating fatty chain of the compact oleic acid shell around PbS creates a charge-transfer resistance for electron transfer, limiting efficient hole injection. Accordingly, to maximize the hole-injection efficiency, the PbS capped with oleic acid were then treated with a solution of tetrabutylammonium iodide (TBAI). The detailed synthetic procedure of the QDs is given in ESI. High resolution transmission electron microcopy (HR-TEM) micrographs reveal that the PbS nanoparticles are highly crystalline with a near-monodisperse distribution and an average diameter of around 2.9 nm ± 0.23 nm (Figs 2 and S1). The UV-vis absorption spectra of PbS QDs in toluene (Fig. 2) exhibits an excitonic band peaking at 909 nm, corresponding to a confined bandgap of 1.37 eV in agreement with the calculation following the empirical equation of Iwan Moreels and co-workers^31^. Although not significantly altering the size of the QDs, treatment with iodide red-shift the absorption and emission absorption and emission maxima (Figs S2 and S3).

The photoluminescence spectra of PbS QDs, recorded in solution, show a narrow Gaussian-shaped band (Figs 2 and S3). The QDs treated with TBAI exhibit an intense emission band four times more intense than that of the QDs capped only by OA (Fig. S3). The higher emission quantum yield of PbS-TBAI is consistent with a lower defect concentration on PbS owing to passivation by the iodide trap. In this study, the QDs were immobilized on NiO surface following the linker-assisted method rather than *in-situ* methods, because the former enables the use of well-defined QDs in terms of size, crystallinity and composition (see Scheme S1). Moreover, the traps can be healed by using suitable post-synthetic treatments^4^,^5^. Screen-printed nanocrystalline NiO films were first coated by MPA by immersion of the electrode into a solution of the latter. It is important to note that dipping the bare NiO electrode (without MPA) into the PbS colloidal solution results in low PbS loading as evidenced by the weak color change of the NiO film. This behavior may be attributed to the inefficient wetting of the hydrophilic NiO network by the fatty hydrophobic PbS QDs. In contrast, the initial coverage of NiO surface by MPA prior to PbS deposition not only decreases the hydrophilicity of the NiO surface, but also improves the surface chemical affinity to PbS owing to the presence of free thiol groups pointing upwards from the surface. Indeed, the MPA-modified NiO electrode rapidly turns dark after immersion into the PbS colloidal solution. The PbS-coated NiO electrode was then treated with a solution of cetyl trimethyl ammonium bromide (CTAB) to substitute additional OA ligands with halides. This treatment decreases the hydrophobic volume of the organic shell around the QD and reduces the mean distance with NiO–an effect favorable for hole injection rate–and also allows for a more densely packed organization of the QDs upon the NiO surface. A second dipping of the already PbS coated NiO film into the PbS solution further increased the loading of the QDs. The replacement of CTAB by TBAI also improves the PCE of the resulting cells but to a lower extent. The reason for this is not yet understood, but can be due to a synergetic effect of dual halide treatment (I^−^ + Br^−^) upon the trap passivation efficiency. Overall, these successive steps are summarized on Scheme S1 and have been shown to be particularly important for reaching a high coverage of the NiO surface by the QDs. It is indeed well-accepted that the QD-loading is a crucial step for determining the final performances of p-QDSSCs^6^,^7^. The PbS-modified NiO films were subsequently investigated by ATR-IR to elucidate the impact of TBAI and CTAB treatments on the ligand shell around PbS (Figs 3 and S4).

The steadily decreasing intensity stretching and bending bands of the carboxylic acid in the region 1400–1550 cm^−1^ and the decrease of the CH stretching vibrations in the region 2850–2925 cm^−1^ upon treatment by TBAI followed by CTAB are clear indications of a steady decrease of the OA concentration around PbS by substitution of OA ligands by iodide and bromide anions (Fig. S4). It is worthy of note that the strong stretching vibration of the carbonyl group at 1700 cm^−1^ is barely visible in the IR spectra, indicating that there are no free or few carboxylic acid groups originating from OA or MPA, as these are all coordinated to lead or nickel atoms. The NiO nanoparticles were then scratched from the electrodes and imaged by HR-TEM (Figs 4 and S5). The presence of PbS as a highly dense monolayer on the NiO surface can be clearly distinguished from the characteristic lattice plane spacing and electronic diffraction pattern of each material. The packing density of PbS nanocrystals is higher after the second immersion of NiO electrode into PbS solution compared to the first dipping cycle (Figs 4 and S5).

**Figure 1:**
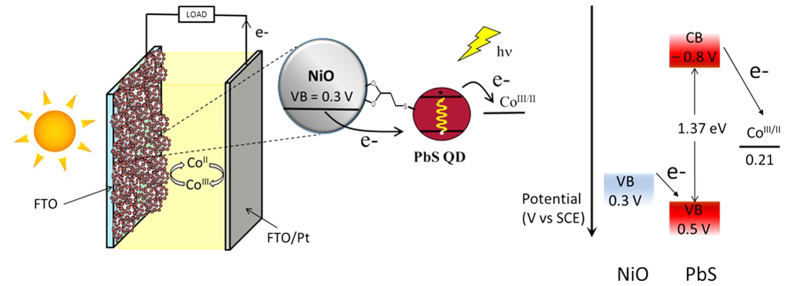
Operation principle of a NiO based p-QDSSC and band positions of the pertinent states of the cell components.

**Figure 2:**
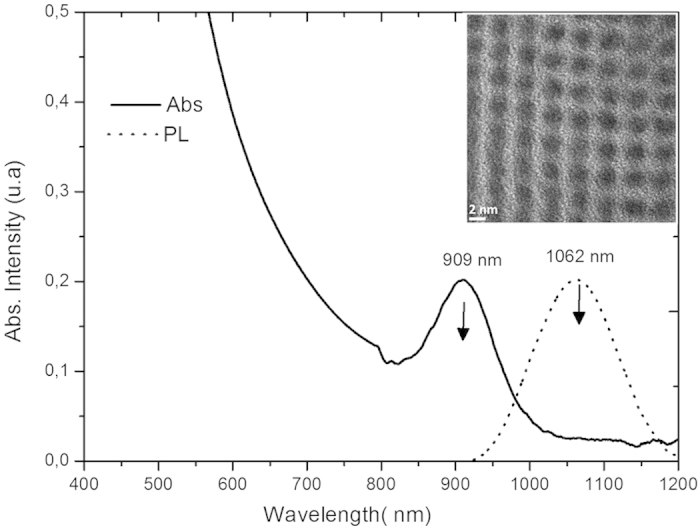
Absorption (straight line) and emission (dashed line) spectra of PbS-OA QDs recorded in toluene and HR-TEM image of these nanocrystals (insert).

**Figure 3:**
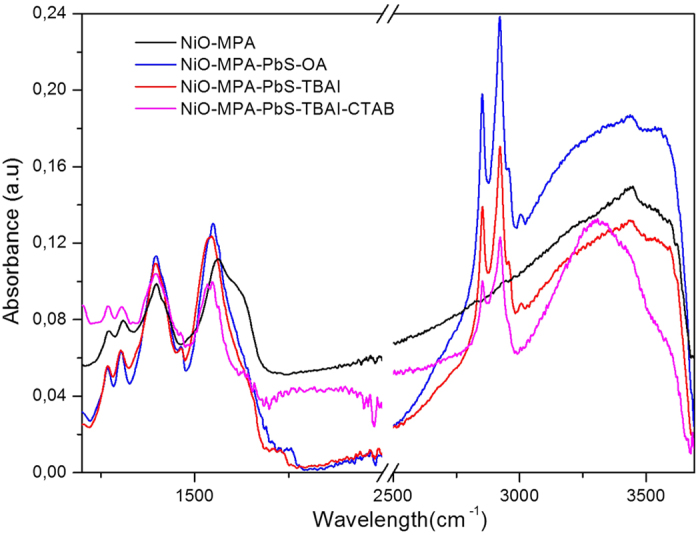
Attenuated total reflectance infrared (ATR-IR) spectra of NiO film coated with MPA (black), MPA-PbS-OA (blue), MPA-PbS-TBAI (red) and MPA-PbS-TBAI-CTAB (magenta).

**Figure 4:**
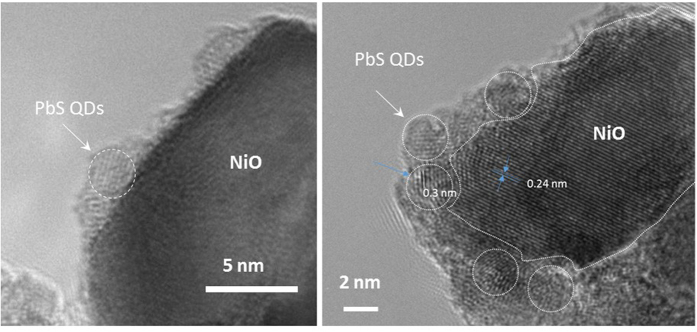
HR-TEM images of the NiO particles coated with PbS-TBAI after one (left) and after two coating cycles (right). The PbS QDs are delimited by a circle for an easier viewing.

Having deposited the PbS quantum dots on the Pt electrode, a cyclic voltammogram was recorded to access the conduction and valence band potentials as already reported^32^. The valence band (VB) edge of PbS QDs is more positive (0.5 V vs SCE) than that of NiO (0.3 V vs SCE)^33^, thus ensuring a 0.2 eV driving force for hole injection (Fig. 1). In addition, the conduction band (CB) of PbS is more negative (−0.8 V vs SCE) than the redox couple Co^III/II^ potential (0.21 V vs SCE)^34^, indicating that the reduction of the electrolyte is highly exergonic. The resulting electrochemical bandgap (1.3 eV) is in good agreement with that estimated from the electronic spectra (1.37 eV).

### Photoelectrochemical study

**Figure 5:**
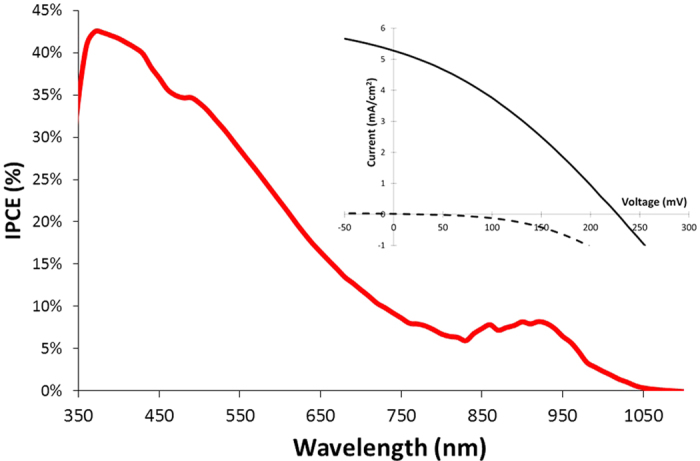
IPCE spectrum of NiO solar cell coated twice with PbS-TBAI quantum dots. Insert: current/voltage characteristics of the cell recorded under AM1.5 (straight line) under the dark (dashed line).

The solar cells were then fabricated by assembling the NiO photocathode with a Pt counter electrode sandwiching an electrolyte film composed of two cobalt complexes as the redox shuttle (see structure Fig. S6). Although the cobalt electrolyte is rarely used in QDSSCs^35^, we have selected it for the several advantages that it brings to bear for these systems. First, PbS QDs are known to be unstable with iodide and polysulfide electrolytes, whereas they are chemically inert with cobalt polypyridine complexes such as those used here. Second, cobalt redox mediators allow higher photovoltages to be reached in both p-DSSCs and n-DSSCs compared to iodide and polysulfide electrolytes^3^,^34^. Third, their compatibility with the TiO_2_ photoanode permits us to envision their use in the fabrication of tandem DSSCs. Finally, the cobalt complexes can be used as catalysts for proton reduction, an ingredient in the route towards photocatalysis^36^,^37^. The photovoltaic performances of the p-QDSSCs were measured under simulated solar light (AM 1.5) along with those of a cell sensitized with an active molecular dye^38^ (see structure P1 Fig. S6) for comparison (Table 1 and Fig. S7). The cells made with the NiO film coated only with the as-prepared solution of PbS-OA gives moderate performances with particularly low Jsc, likely due to the low coverage of the NiO surface. In addition, the presence of numerous traps in the QDs and the thick saturated fatty chain of OA ligands around PbS limits the hole injection quantum yield^6^,^7^,^21^,^27^. The treatment of QDs by TBAI positively impacts the performances considering that the J_SC_ and V_OC_ of the cell were both enhanced, consistent with a higher injection quantum yield and lower interfacial charge recombination with the electrolyte, as can be seen in the electrochemical impedance spectroscopy measurements below. Finally, a subsequent second coating of the NiO film by the PbS solution further enhances the photocurrent density to 5.27 mA/cm^2^. Most importantly, the photoaction spectrum shows that photocurrent is produced even at long wavelengths of up to 1000 nm (Fig. 5), a feature that has never been previously observed in NiO based p-DSSC with any type of photosensitizer.

Interestingly, the solar cell sensitized with the PbS quantum dots outperforms that based on the molecular dye P1 (structure shown in Fig. S6), even if the latter is used with the iodide/triodide electrolyte with the composition giving the best photovoltaic performances^39^. The P1 dye exhibits an even lower PCE with the cobalt electrolyte, owing to the rapid charge recombination rate constant precluding the reduction of the Co(III) redox mediator, the latter being a slow electron acceptor^34^,^40^. The high photocurrent recorded in PbS p-QDSSC is an indirect indication that the charge separated state is significantly long-lived, and certainly much longer than the lifetime with the P1 molecular dye, for which Jsc and Voc are much lower.

Overall, these results demonstrate that hole injection in NiO is significantly efficient from a low bandgap QD. Consequently, this study supports the capacity of these photocathodes for harvesting the low-energy part of solar spectrum, a favorable feature for complementing the photoactivity domain of a regular n-type photoanode based on sensitized TiO_2_ films. Preliminary tests indicate that the stability of the cell is satisfactory, considering that we have observed almost no drop of the photovoltaic performances of the sealed cells over the course of 500 hours stored in laboratory lightning (Fig. S8). To shed some light on the effect of the different treatments, the solar cells were investigated by means of electrochemical impedance spectroscopy (EIS) measurements. The Nyquist plots feature two semicircles: a high-frequency loop corresponds to the charge transfer resistance at the counter-electrode (R_CE_) and a middle frequency semicircle corresponds to the charge transfer resistance (R_REC_) across the NiO/QD/electrolyte interface (Fig. S9)^41^. The interfacial NiO/electrolyte charge transfer resistance increases from the NiO film coated with QDs treated by TBAI compared to that from the as-prepared QDs (PbS-OA) since R_REC_ passes from 550 Ω to 1180 Ω respectively. R_REC_ further increases after the second PbS-TBAI coating, reaching resistances of 15650 Ω. On the other hand, the hole lifetime in NiO (τ_h+_) consistently increases in the cell coated with PbS-OA (0.026 s) to PbS-TBAI (0.28 s) to the cell coated twice with PbS-TBAI (3.1 s) (Table S1). Collectively, these results support that the degree of surface coverage of NiO surface by the QDs increases as the PbS ligand shell is replaced from OA molecules to halide ions, an effect that enables a closer packing of the QDs, in turn creating a passivating physical barrier to the redox mediator approach and enhances the absorption cross-section.

## Conclusion

In this study, we have developped a procedure to graft a large amount of PbS quantum dots (QDs) on the surface of a nanostructured NiO photocathode. This study demonstrates that the low bandgap quantum dot PbS can act as efficient sensitizer in p-DSSC, exhibiting both promising photostability and measured PCE values that exceed those of the highly performing P1 molecular dye. Importantly, photocurrent is produced into the IR range. This study highlights that low bandgap QDs are promising materials for p-type NiO based solar cells, even with the use of a slow redox mediator such as a cobalt tris-bipyridine complex. This finding that QD-sensitized NiO based-photocathodes can exploit low-energy photons strongly evidences their potential to be integated in tandem cells in order to enhance the harvesting of sunlight for sustainable energy production.

## Methods

### Materials synthesis

The preparation of colloidal quantum dots (PbS-OA) are described in the ESI according to the standard method from lead oleate and bis(trimethylsilyl) by the hot injection technique^9^,^10^.

### Solar cell fabrication

Conductive glass substrates (F-doped SnO_2_) were purchased from Solaronix (TEC15, sheet resistance 15 Ω/square). Conductive glass substrates were successively cleaned by sonication in soapy water, then ethanol for 10 min before being fired at 450 °C for 30 min. Once cooled down to room temperature, FTO plates were rinsed with ethanol and dried in ambient air.

NiO compact layer was prepared by spin-coating onto the clean FTO substrates with a 0.5 M nickel acetate solution containing 0.5 M ethanolamine in methoxyethanol at 2000 rpm for 30s followed by thermal treatment at 500 °C for 0.5 h. The thickness of the NiO dense layer was measured by SEM on sliced substrates (30 ± 5 nm). Then, NiO mesoporous layer was deposited on the dense layer by screen-printing with a semi-automatic screen printer. The NiO screen-printing paste was composed of 3 g of NiO nanopowder (Inframat) suspended in 10 mL of distilled ethanol and ball-milled (500 rpm) for 24 h. The resulting slurry was mixed in a round-bottom flask with 10 ml of 10 wt% ethanolic ethyl cellulose (Sigma Aldrich) solution and 20 ml terpineol, followed by slow ethanol removal by rotary evaporation. Two layers of mesoporous NiO were subsequently screen printed, with intermediate drying of the films. The dried films were finally first calcined in air at 400 °C for 0.5 h. The prepared NiO electrodes (3 μm thick) were soaked in a solution of nickel acetate in ethanol (20 mM) with 1% triethanolamine for 30 min at 60 °C followed by ethanol rinsing and drying in air. The cells were then fired at 200 °C for 30 min. The thickness of the resulting NiO films was measured with a DEKTAK Profilometer (~3 μm).

NiO electrodes were treated with 35% MPA (volume) in acetonitrile for 3 min and dried in air. The films were then immersed in PbS QDs solution for 12 h. The NiO-PbS films were rinsed with octane and dried in air and then treated with a solution of CTAB (cetyltrimethylammonium bromide) in methanol (10 mg/mL) for 5 min and followed by rinsing with methanol to remove excess CTAB. This process was repeated twice.

The cobalt electrolyte was composed of 0.1 M [Co^II^(dtb-bpy)_3_](PF_6_)_2_, 0.1 M [Co^III^(dtb-bpy)_3_](PF_6_)_3_ where dtb-bpy stands for 4,4′-diterbutyl-2,2′-bipyridine (see Fig. S6), and 0.1 M LiClO_4_ in propylene carbonate. The iodide electrolyte for solar cell sensitized with **P1** dye (see Fig. S6) was composed of [LiI] = 1 M and [I_2_] = 0.1 M in acetonitrile. Counter electrodes were prepared by chemical deposition of platinum from hexachloroplatinic acid in distilled isopropanol (10 mg per mL) on FTO substrates (Solaronix, TEC 7). The two electrodes were assembled on top of each other using a thin transparent film of Surlyn polymer (DuPont, 25 μm) as a spacer. The empty cell was tightly held, and the edges were heated to 110 °C to seal the two electrodes together. A drop of electrolyte was introduced through a predrilled hole in the counter electrode by vacuum backfilling, and was sealed afterward with a thin pastille of glass and Surlyn. The cell had an active area of 0.25 cm^2^.

### Characterizations

UV-Visible absorption spectra were recorded on a *UV-2401PC Shimadzu* spectrophotometer. Fluorescence spectra were recorded on a Horiba Jobin-Yvon *SPEX Fluoromax* fluorimeter. Infrared spectra (ATR) were recorded on a *BRUKER Vector 22* spectrometer; frequencies are reported in cm^−1^. Transmission electron microscopy (TEM) was performed with a Hitachi HF2000 (cold FEG, accelerating voltage 100 kV, point resolution 0.23 nm).

Electrochemical impedance spectroscopy (EIS) experiments were carried out on the DSSC to access the charge transfer resistance and chemical capacitance of the various interfacial processes with a Potentiostat model VSP from Bio-Logic Science Instruments. The impedance spectra of the solar cells were recorded in the dark and under 1 sun illumination at V_oc_ in the frequency range of 100 KHz to 50 mHz. The photocathode was connected to the working electrode and the anode to the counter and reference electrodes.

The current-voltage characteristics were determined by applying an external potential bias to the cell and measuring the photocurrent using a Keithley model 2400 digital source meter. The solar simulator is an Oriel Lamp calibrated at 100 mW/cm^2^ with a Si reference solar cell to determine the optical power.

## Additional Information

**How to cite this article**: Raissi, M. *et al.* Infra-red photoresponse of mesoscopic NiO-based solar cells sensitized with PbS quantum dot. *Sci. Rep.* **6**, 24908; doi: 10.1038/srep24908 (2016).

## Supplementary Material

Supplementary Information

## Figures and Tables

**Table 1:** Photovoltaic performances of NiO-based p-DSSCs recorded under simulated solar light AM1.5 (100 mW/cm^2^).

NiO sensitized with	V_OC_(mV)	J_SC_(mA.cm^−^^2^)	ff (%)	η (%)
PbS-OA^c^	147	0.90	32	0.04
PbS-TBAI^c^	240	3.50	34	0.28
PbS-(TBAI)_2_^a^^,c^	227	5.27	33	0.40
P1^b^	88	4.27	31	0.12
P1^c^	107	1.07	26	0.020

^a^NiO film coated twice with QD.

^b^I_3_^−^/I^−^ electrolyte.

^c^Cobalt electrolyte.
