# Supplementary material for: Infra-red photoresponse of mesoscopic NiO-based solar cells sensitized with PbS quantum dot
Source: Sci Rep. 2016 Apr 29;6:24908. doi: 10.1038/srep24908 (PMC4850376; doi:10.1038/srep24908)
Supplement: Supplementary Information [file srep24908-s1.pdf]

Supporting Information Materials for "Infra-red photoresponse of mesoscopic NiO-based solar cells sensitized with PbS quantum dot"

Mahfoudh Raissi,[a] Yann Pellegrin,[a] Stéphane Jobic,[b] Mohammed Boujtita,[a] Fabrice Odobel*[a]

[a]CEISAM, Chimie Et Interdisciplinarité, Synthèse, Analyse, Modélisation
CNRS, UMR CNRS 6230, UFR des Sciences et des Techniques
2, rue de la Houssinière - BP 92208; 44322 NANTES Cedex 3 (France)
E-mail: Fabrice.Odobel@univ-nantes.fr
[b] Institut des Matériaux Jean Rouxel, Université de Nantes, CNRS, 2, rue de la Houssinière 44322 NANTES Cedex 3 (France)

Preparation of PbS quantum dots

Colloidal quantum dots (PbS OA) were synthesized by a standard method[1]. Typically, in a first Schlenk tube was introduced a mixture of 0.210 ml of bis(trimethylsilyl) sulphide (TMS, synthesis grade) in 1-octadecene (10 ml). The solution was degassed under argon at room temperature for 2h. In second Schlenk tube, PbO (0.45g) was suspended in 15 ml of 1-octadecene and 1.5 ml of oleic acid was added. The mixture was degassed and heated at 95°C under Ar. After 1h, the solution became colourless (formation of lead oleate). Then, the temperature of solution was increased to 120°C for 2 hours under argon. Finally, the Schlenk temperature was decreased at 110°C and the TMS/octadecene mixture was injected quickly and after 2 min of reaction, the solution was cooled down to room temperature. The PbS QDs were precipitated with 50 mL of acetone and centrifuged. The supernatant was removed and the QDs were dispersed in toluene. Then, the QDs were precipitated again in acetone (30 mL) and centrifuged. Finally, the nanocrystals were dispersed in toluene (50 mg/ml).

PbS-OA were treated with tetrabutylammonium iodide (TBAI) by addition of 0.5 mL (50 mg/ml) of PbS-OA solution (5 ml) and stirred at room temperature for 15 min. 10 ml of ethanol were added to precipitate the QDs and the solution was centrifuged. PbS-TBAI were finally dispersed in octane (30 mg/ml).

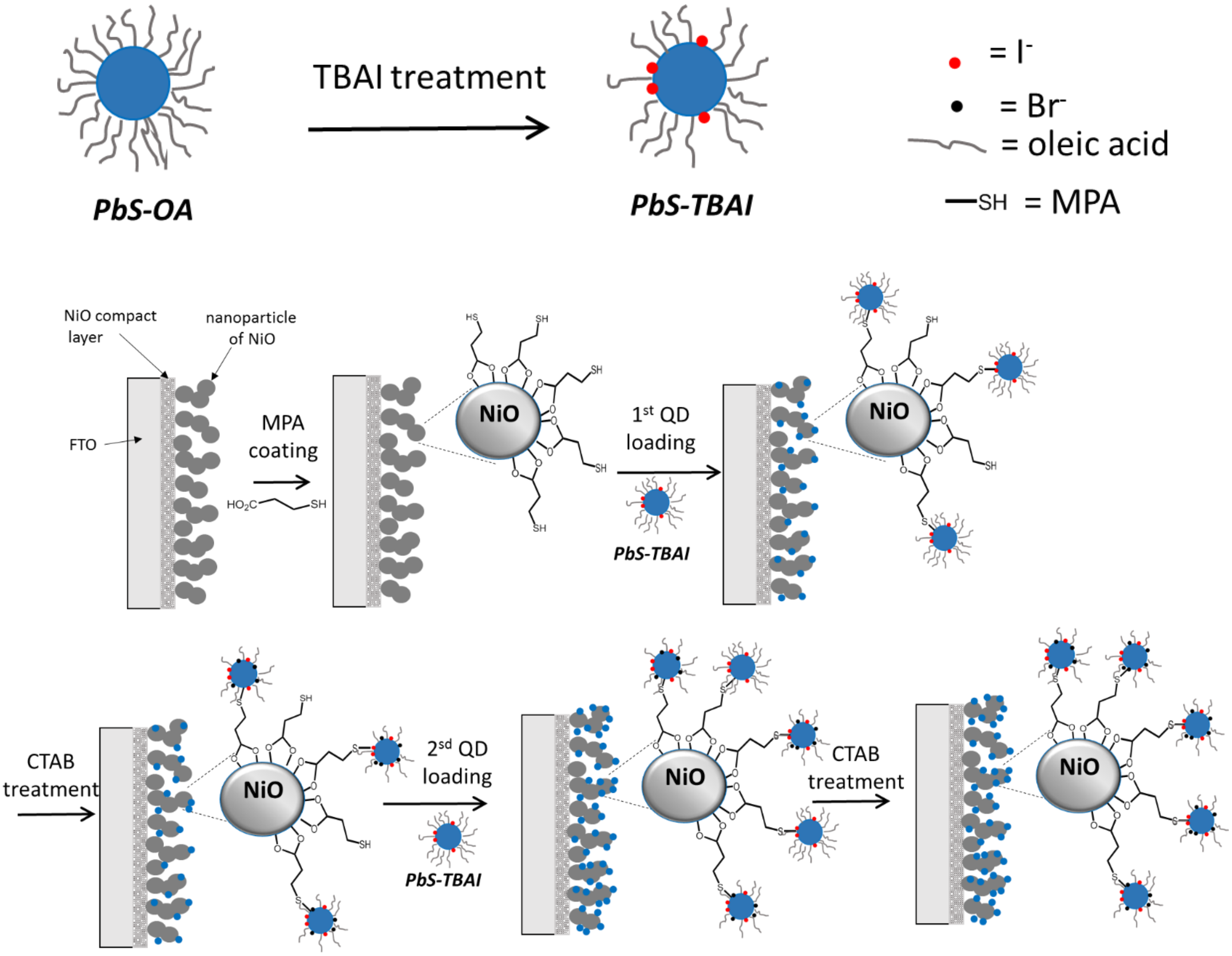

Scheme S1. Cartoon schematically showing the different steps of the photocathode fabrication.

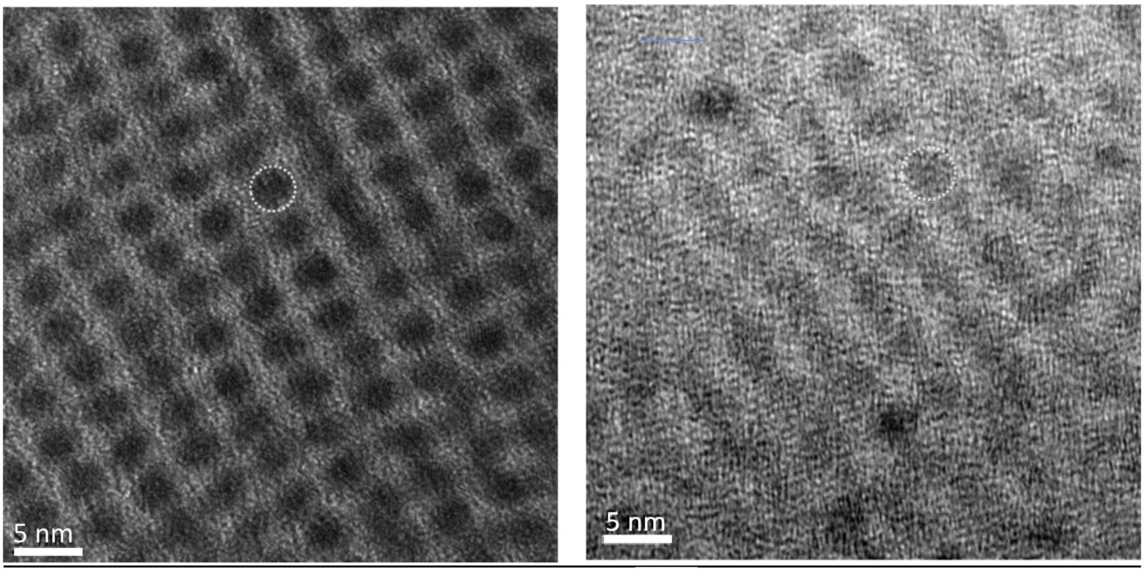

Figure S1. HR-TEM image of PbS-OA quantum dot (left, average diameter: 2.9 nm) and PbS-OA treated with TBAI (right, average diameter: 3.0 nm).

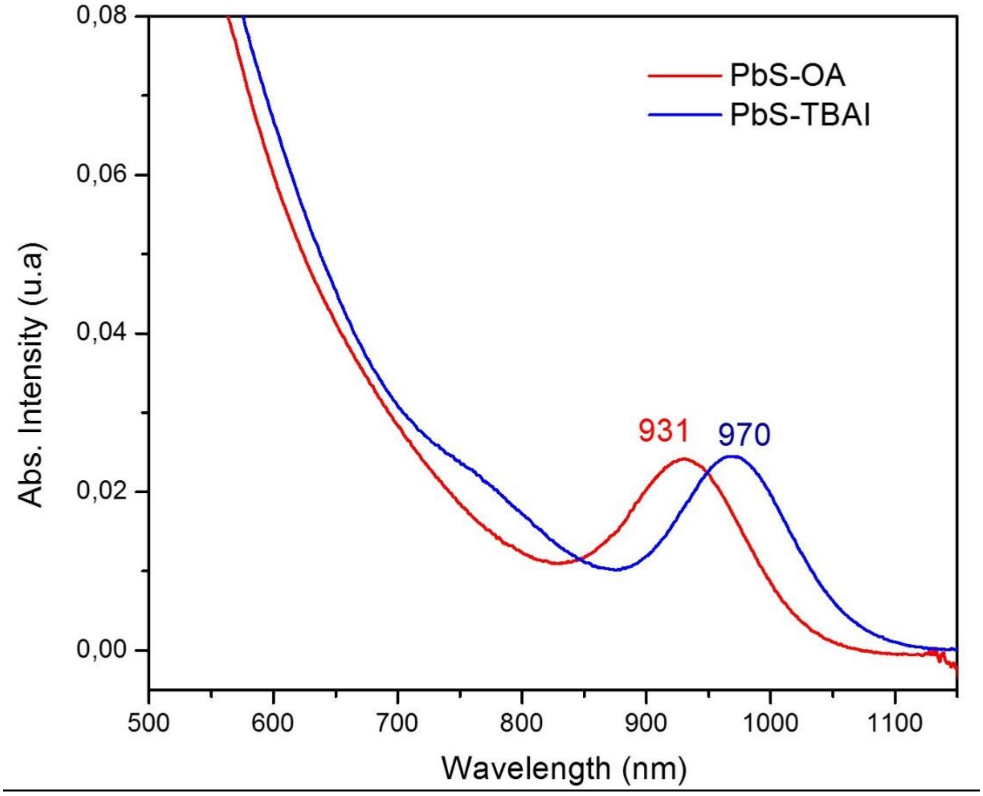

Figure S2. Absorption spectra of PbS-OA QDs (red curve) and PbS-TBAI QDs (blue curve) recorded in toluene and octane solution respectively.

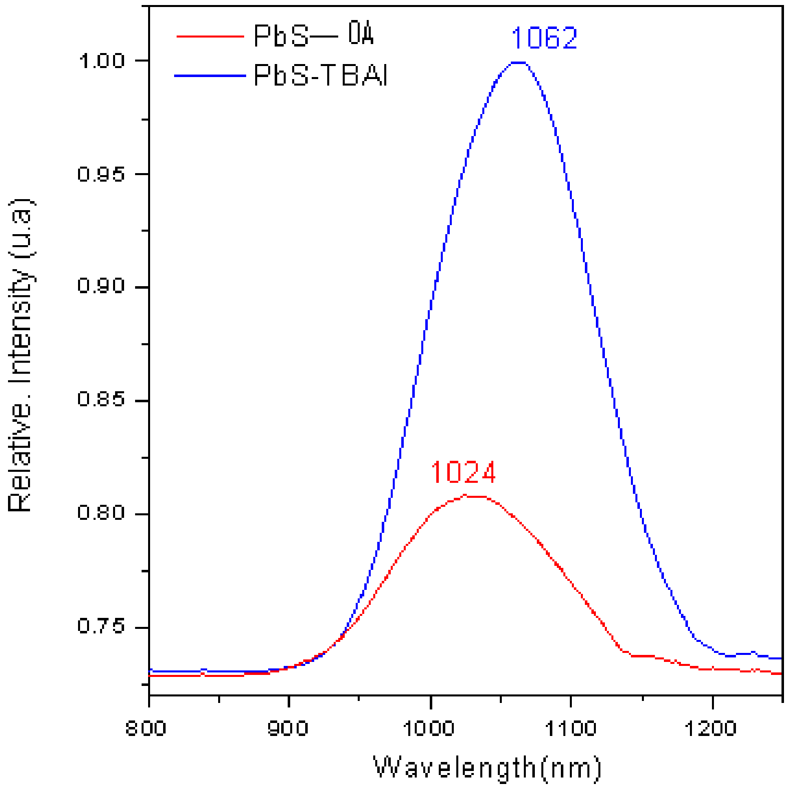

Figure S3. Photoluminescence spectra of PbS-OA quantum dot (red curve) and PbS-OA after treatment with TBAI (blue curve) in toluene and octane solution respectively recorded with isoabsorbance solution at 950 nm.

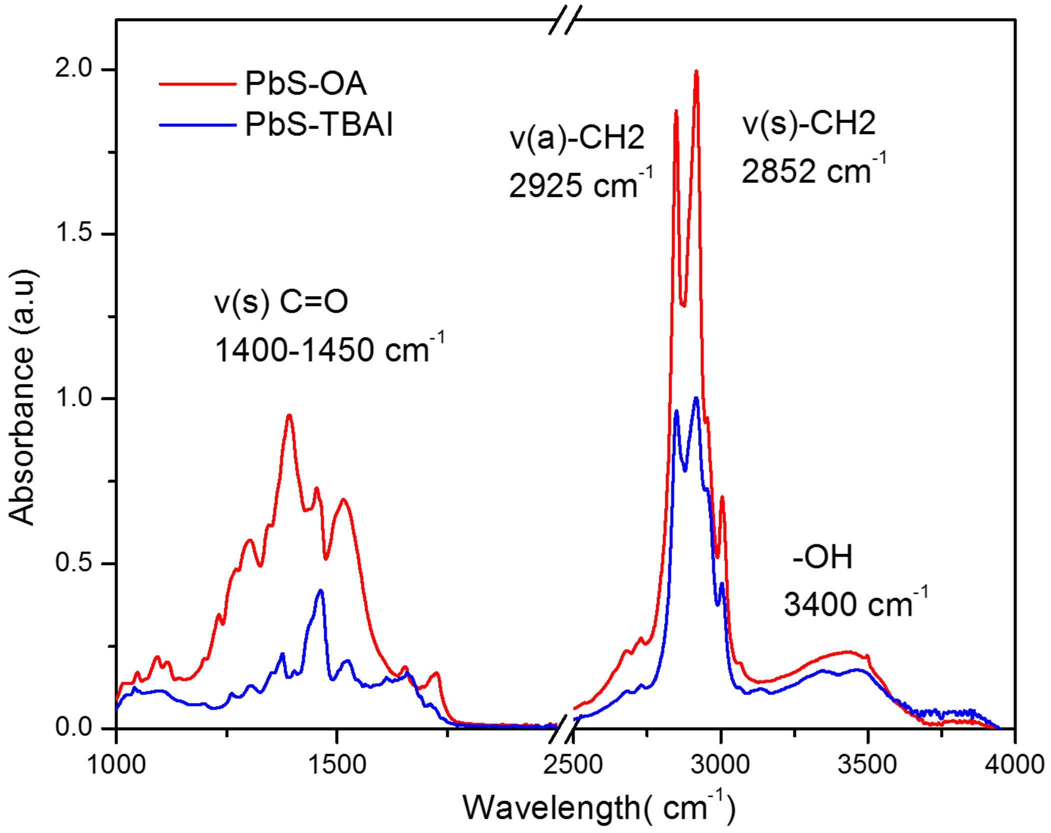

Figure S4. Infrared spectra of PbS-OA quantum dot untreated (red curve) and PbS-TBAI after treatment with TBAI solution (blue curve).

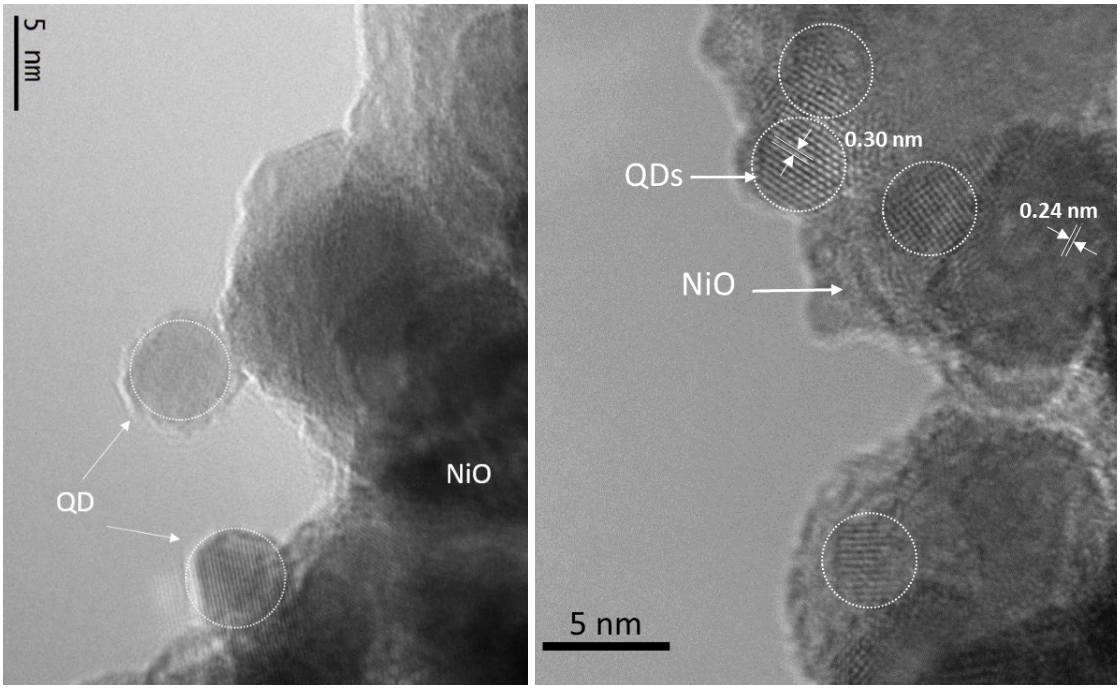

Figure S5: TEM images of PbS-OA on NiO (left) and PbS treated with TBAI on NiO (right).

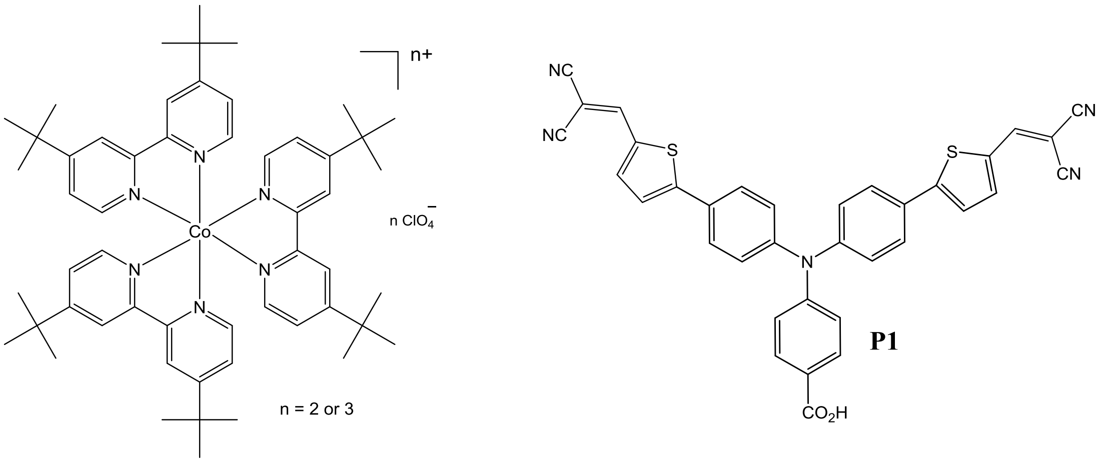

Figure S6. Structure of the cobalt complexes used as redox shuttle and of the molecular dye **P1**.

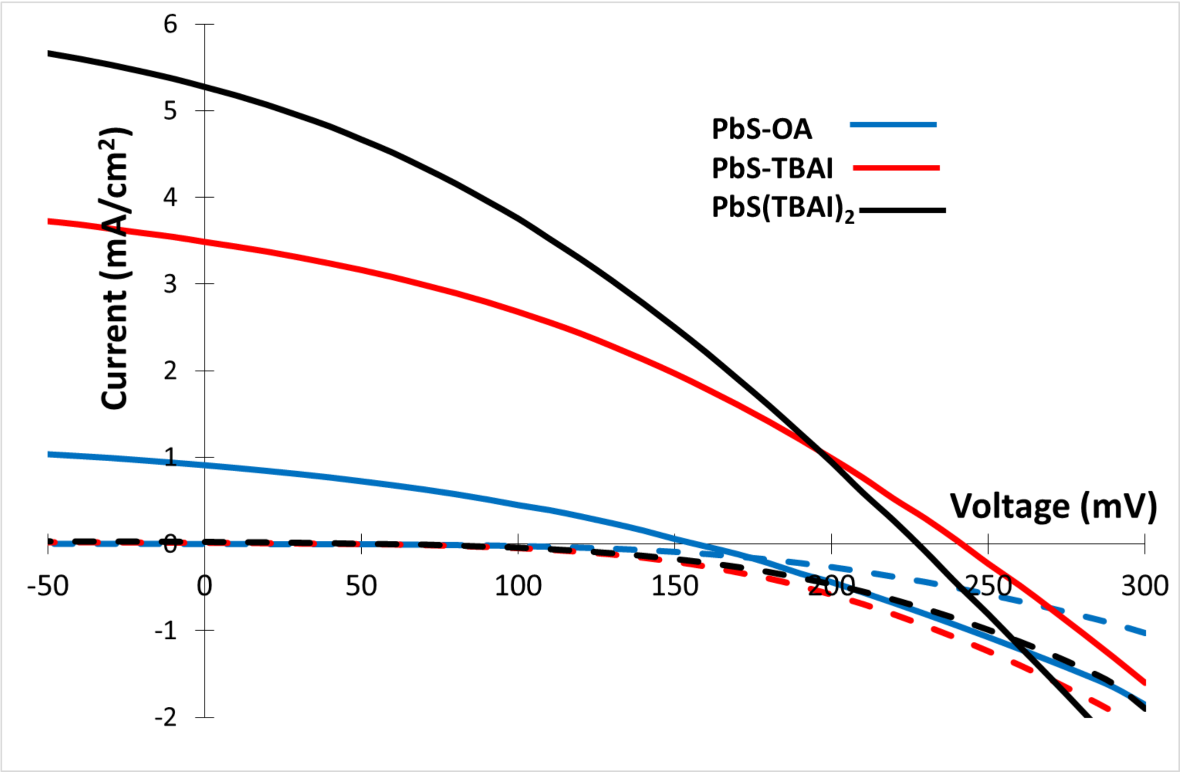

Figure S7. Current/voltage characteristics of different PbS sensitized solar cells recorded under simulated AM1.5 (straight line) and under the dark (dashed line).

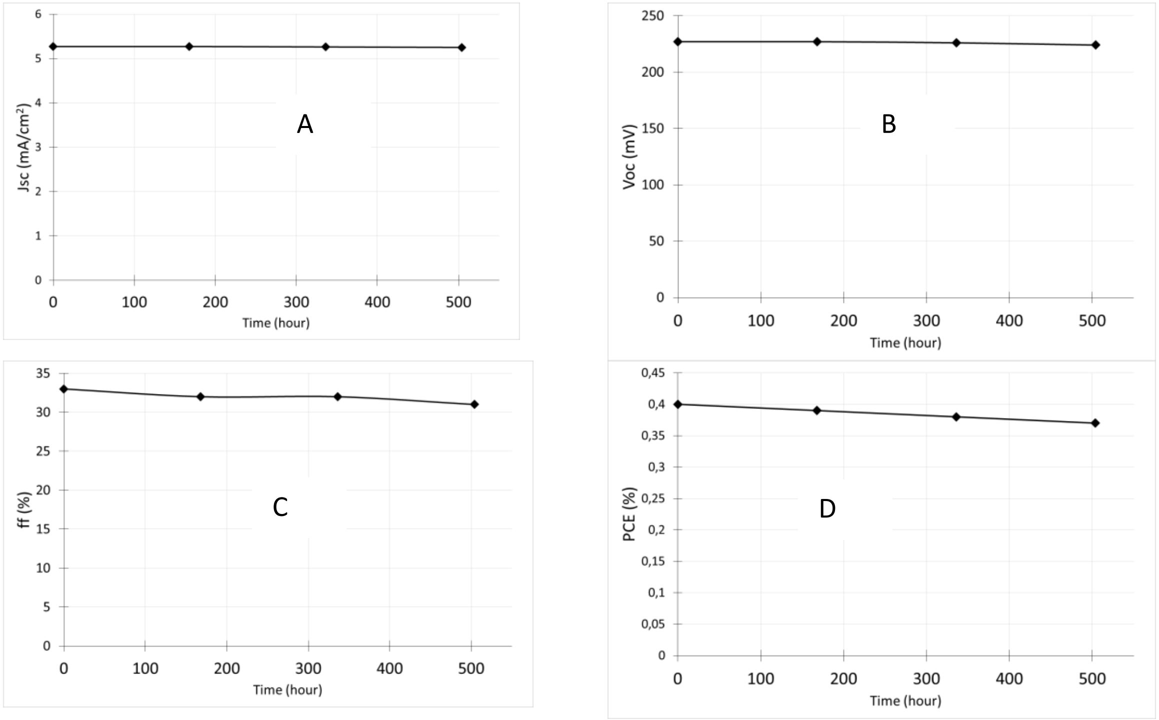

Fig.S8. Evolution of the solar cells metrics of the p-QDSSCs sensitized with NiO film coated twice with PbS-TBAI and upon aging on the laboratory bench. A) Jsc, B) Voc, C) ff and D) PCE.

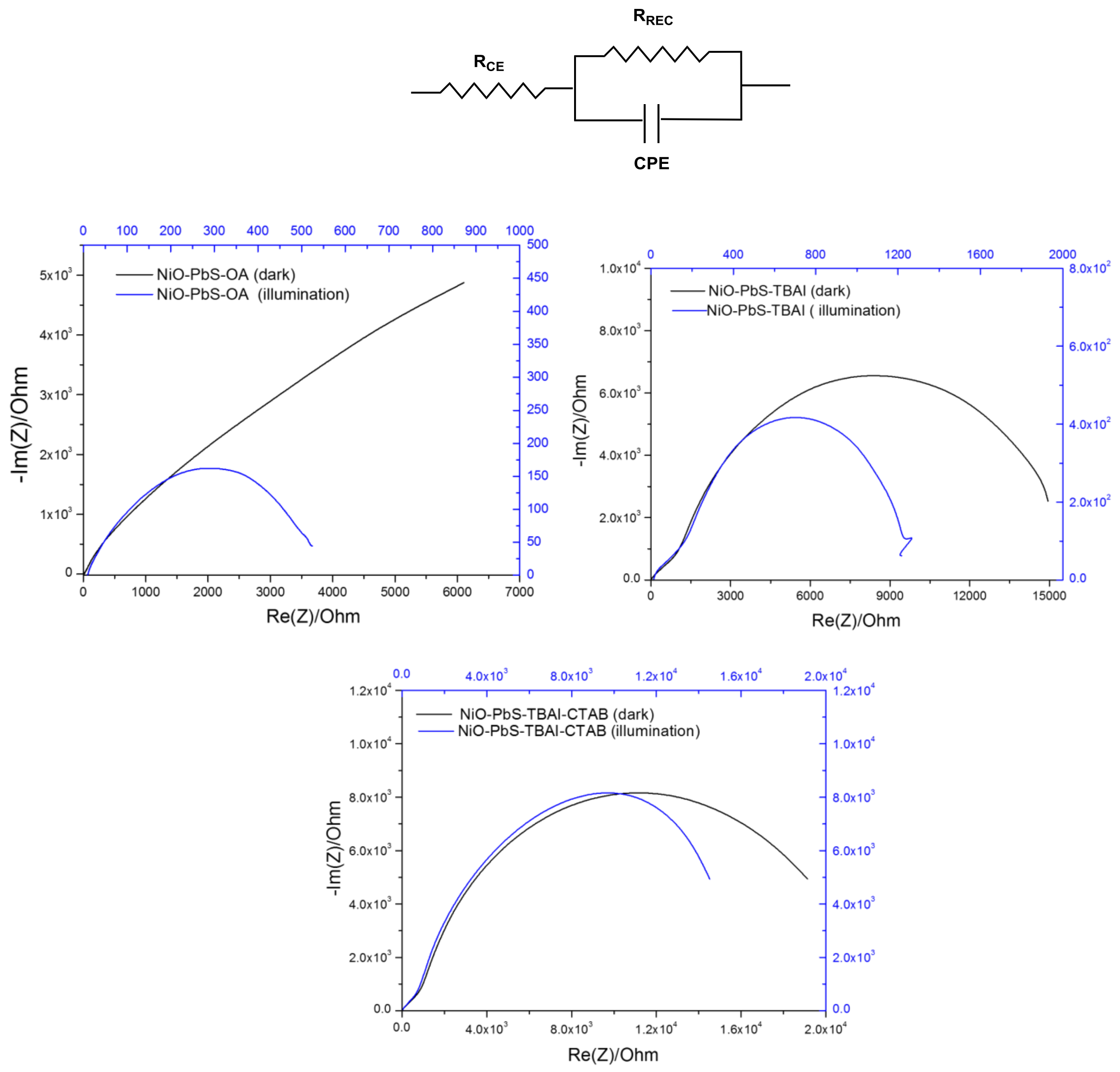

Figure S9: Nyquist plots of the cells recorded in the dark (black line) and under illumination (blue line) sensitized with PbS-OA, PbS-TBAI and PbS-TBAI +CTAB+PbS-TBAI. The equivalent electric circuit is shown on the top.

Table S1. Values of capacitances (C), charge transfer resistances (R_{REC}) and hole lifetime (τ_{h+}) of the solar cells based coated with MPA-PbS-OA, MPA-PbS-TBAI and MPA-PbS-TBAI+CTAB+PbS-TBAI recorded in dark and under illumination.

In dark	NiO-MPA-PbS-OA	NiO-MPA-PbS-TBAI	NiO-PbS-TBAI +CTAB+PbS-TBAI
C (F)	$1.94x10^{-4}$	$1.54x10^{-4}$	$1.94x10^{-4}$
R_{REC} (Ω)	13720	17705	20655

Under illumination	NiO-PbS-OA	NiO-PbS-TBAI	NiO-PbS-TBAI +CTAB+PbS-TBAI
C (F)	$4.78x10^{-5}$	$2.34x10^{-4}$	$2.01x10^{-4}$
R_{REC} (Ω)	550	1180	15650
τ_{h+} (s)	0.026	0.28	3.1
